# Malaria epidemiological research in the Republic of Congo

**DOI:** 10.1186/s12936-016-1617-7

**Published:** 2016-12-23

**Authors:** Felix Koukouikila-Koussounda, Francine Ntoumi

**Affiliations:** 1 Fondation Congolaise Pour la Recherche Médicale, Villa D6, WHO AFRO Campus, Brazzaville, Republic of Congo; 2 Faculty of Sciences and Techniques, University Marien Ngouabi, Brazzaville, Republic of Congo; 3 Institute for Tropical Medicine, University of Tübingen, Tübingen, Germany

**Keywords:** Malaria, *Plasmodium falciparum*, Review, Health research, Republic of Congo

## Abstract

**Background:**

Reliable and comprehensive information on the burden of malaria is critical for guiding national and international efforts in malaria control. The purpose of this review is to provide an overview of published data and available information on malaria resulting from field studies/investigations conducted in the Republic of Congo (RoC) from 1992 to 2015, as baseline for assisting public health authorities and researchers to define future research priorities as well as interventions.

**Methods:**

This review considers data from peer-reviewed articles and information from the National Malaria Control Programme reports, based on field investigations or samples collected from 1992 to 2015. Peer-reviewed papers were searched throughout online bibliographic databases PubMed, HINARI and Google Scholar using the following terms: “malaria”, “Congo”, “Brazzaville”, “prevalence”, “antimalarial”, “efficacy”, “falciparum”, “genetic”, “diversity”. Original articles and reviews were included and selection of relevant papers was made.

**Results:**

Twenty-eight published articles were included in this review and two additional records from the National Malaria Control Programme were also considered. The majority of studies were conducted in Brazzaville and Pointe-Noire.

**Conclusion:**

The present systematic review reveals that number of studies have been conducted in the RoC with regard to malaria. However, their results cannot formally be generalized at the country level. This suggests a need for implementing regular multisite investigations and surveys that may be representative of the country, calling for the support and lead of the Ministry of Health.

## Background

Malaria is a life-threatening disease caused by protozoan parasites of the genus *Plasmodium* that are transmitted to humans through the bites of infected *Anopheles* mosquitoes. Five different *Plasmodium* species have been demonstrated to infect humans: *Plasmodium falciparum*, *Plasmodium vivax*, *Plasmodium ovale*, *Plasmodium malariae* and *Plasmodium knowlesi*. Of these, *P. falciparum* is the most dangerous, with the highest rates of complications and mortality [1].

The scaling-up of interventions has reduced the number of malaria cases and deaths between 2000 and 2015 [2–4]. In 2000, the World Health Organization (WHO) estimated 262 million cases of malaria globally, leading to 839,000 deaths against 214 million cases and 438,000 deaths in 2015 [2]. Sub-Saharan Africa remains the region with the highest disease burden and accounts for 88 and 90% of the global clinical cases and deaths, respectively [2].

**Fig. 1:**
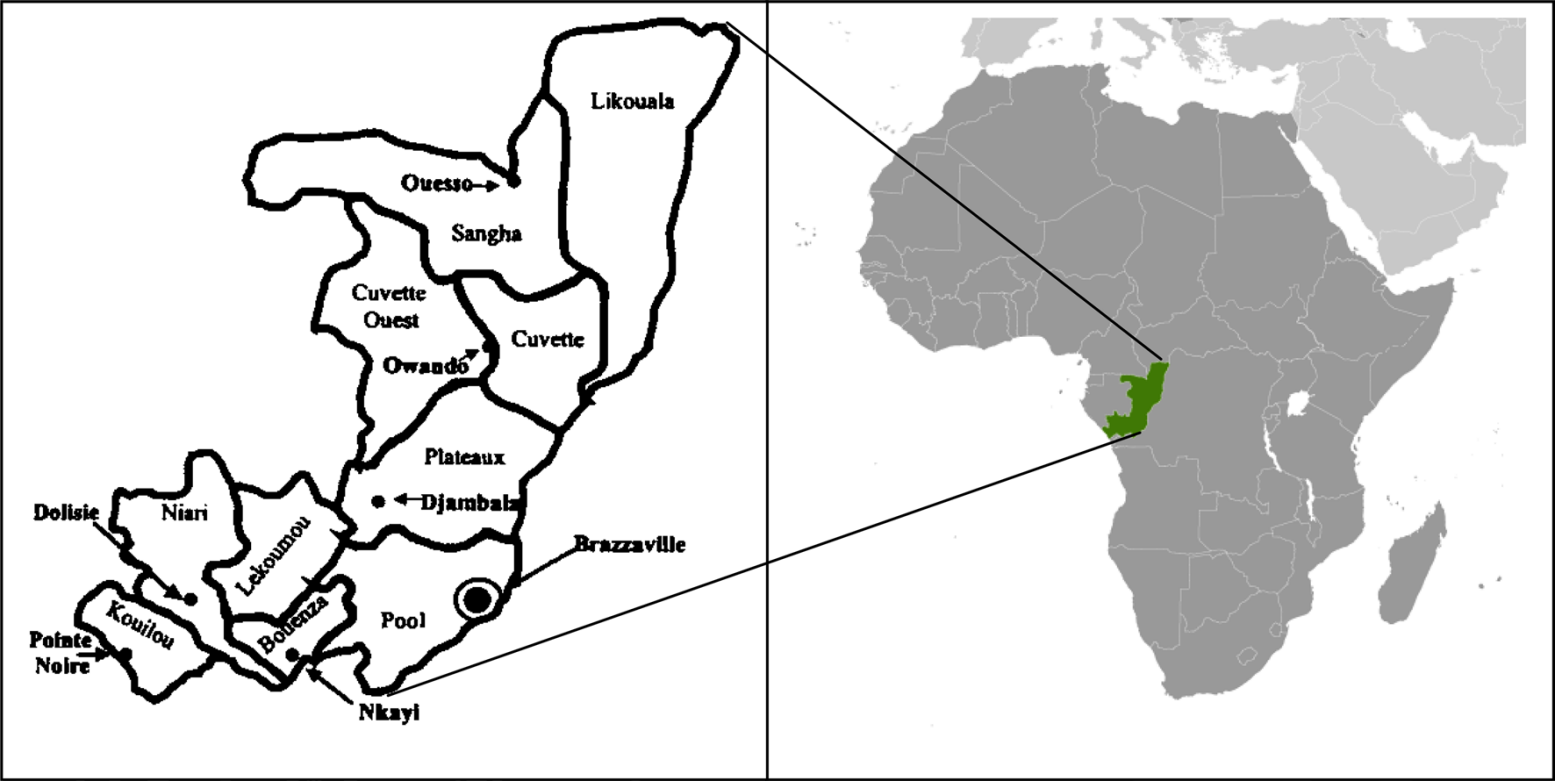
Map of the Republic of Congo (RoC) with its 12 departments

The Republic of Congo (RoC) is one of the 54 countries where malaria transmission is still high [2]. The country is located in the central-western part of sub-Saharan Africa, along the Equator, laying between latitudes 4°N and 5°S, and longitude 11° and 19°E (Fig. 1). It occupies a total surface of 342,000 km^2^ with a population estimated to be 4,800,000 inhabitants, with 61% of its total population living in the two biggest cities, namely Brazzaville and Pointe-Noire [5]. Accordingly, the RoC is one of the most urbanized countries in Africa. The capital, Brazzaville, is located along the Congo River, in the south of the country, immediately across from Kinshasa, the capital of the Democratic Republic of Congo. Since the country is located on the Equator, the climate is consistently humid year-round, with the average day temperature of 25 °C and night between 16 and 21 °C [6]. Two-thirds and one-third of the surface area of RoC is covered by forests and savannah, respectively. The average yearly rainfall ranges from 1100 mm in the south to over 2000 mm in the central and north parts of the country. The rainy season which lasts 9 months, has two rainfall maxima: one in March–May and another in September–November [6]. The dry season is from June to August. A recent entomological survey in RoC jointly conducted by the WHO and the Ministry of Health and Population (MHP) showed that the transmission dynamic of malaria in the country follows two different patterns: (1) a year-round perennial transmission in forest areas, with an estimated entomological inoculation rate (EIR) of 200–1000 infective bite/person/year, and (2) a seasonal transmission in savanna areas where the high transmission period lasts 7–10 months and is directly correlated with the rainfall and the EIR is estimated to be 80–200 infective bites/person/year [7]. Despite considerable efforts and progress in malaria control over the past decades [adoption of artemisinin-based combination therapy (ACT) for the treatment of uncomplicated malaria in 2006, using either artemether–lumefantrine (AL) or artesunate–amodiaquine (ASAQ), use of intermittent preventive treatment with sulfadoxine-pyrimethamine for pregnant women (IPTp-SP), mass distribution of long-lasting insecticide-treated mosquito nets (LLINs) from 2008 to 2012 and free anti-malarial treatment for children aged >15 years since 2008], it remains one of the important public health problems [8]. All over the country, *P. falciparum* is the predominant malaria parasite and *Anopheles gambiae* the predominant mosquito vector. The latest estimations from the National Malaria Control Programme (NMCP) indicate that clinical malaria account for 47.9% of all outpatient consultations in public hospitals, 64.8% of hospital admissions and 18.4% of deaths [5].

Clearly, the country is still struggling with the control phase where expensiveness of the control program as well as its sustainability, resistance of the parasite to anti-malarial drugs and that of vectors to insecticides are some of the challenges. However, some research publications have reported a decrease in clinical malaria prevalence in Southern and Northern sentinel sites of the country [9, 10].

The purpose of this review was to provide an overview of published data and available information from the NMCP of the MHP on malaria situation in RoC including prevalence/incidence trends, vectors, anti-malarial drug efficacy and parasite genetics based on field studies conducted from 1992 to 2015. This may assist in defining future research and intervention priorities.

## Methods

### Search strategy

To collect research data on malaria in RoC, peer-reviewed articles have been retrieved from online bibliographic databases PubMed, HINARI and Google scholar using the following keywords: “malaria”, “Congo”, “Brazzaville”, “1992”, “prevalence”, “antimalarial”, “efficacy”, “falciparum”, “genetic”, “diversity”, “resistance”, “markers”. Reference lists of selected papers were used as leads for identification of additional studies. The Boolean operators “AND”, “OR” and “>” were used to combine two or three terms. In addition, reports from the NMCP were reviewed at the NMCP headquarters in Brazzaville. Predefined medical subject heading (MeSH) was not used to avoid restricting searches.

### Study/document selection and data consideration

**Fig. 2:**
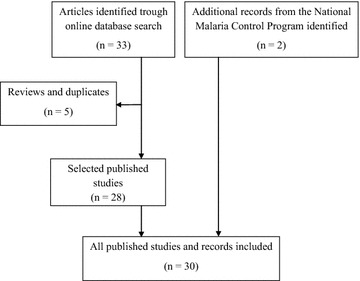
Summary of the search strategy

Studies were included in the review if they explicitly reported on one of the considered aspect of malaria in RoC with: (1) samples collected in the country, (2) a clear description of the methods section (providing the following information: study area, period of sample collection, type of samples collected and study population) and (3) no review articles. An overall of 33 studies were retrieved from the search of peer-reviewed papers, of which five were excluded because of duplications (similar analysis on same samples or study population) or they were review articles. Therefore, 28 studies including full text articles, and short reports written in English or in French were selected and reviewed. Two additional documents from the NMCP were considered (Fig. 2).

## Results

### Malaria parasite prevalence

Published articles included in this review were of samples collected from 1992 to 2015 (Table 1). Of these, nine articles were identified with information on the incidence or the prevalence of malaria parasite infection (Table 2) [9, 11–18]. Most of these studies were performed in children aged ≤15 years. Investigations were conducted in different areas of the country, with most of them being located in Brazzaville and mainly reported the presence of one malaria species, namely *P. falciparum*. There was only one study in which three plasmodial species were detected with a predominance of *P. falciparum* over *P. malariae* (2.9%) and *P. ovale* (0.7%) [11]. Six of the *P. ovale* isolates (two isolates from Brazzaville, two from Pointe-Noire and two from Gamboma) were further characterized by Oguiket et al. using conventional and real-time quantitative PCR methods to discriminate *P. ovale curtisi* and *P. ovale wallikeri*. The two isolates from Brazzaville were identified as *P. ovale curtisi*, while the remaining four isolates were identified as *P. ovale wallikeri* [19]. In another study, a focus was put on providing evidence of transmission of *P. vivax* in RoC [12]. The authors found that 13% of samples, collected in 2007 in Pointe-Noire and tested by enzyme-linked immunosorbent assay, had antibodies to *P. vivax*-specific antigens. Accordingly, they concluded that in conjunction with the frequent reports of travellers returning from western and central Africa with diagnosed *P. vivax* infections, their findings make an argument for the presence and continued transmission of *P. vivax* in RoC. How and where precisely is still unclear and deserves to be further investigated.

**Table 1:** Summary of published articles included in the review

References	Period of sample collection	Year of publication	Study area	Age of participants
Chandenier et al. [20]	1993	1995	Brazzaville, Niari, Kouilou, Pool	6–10 years
Durand et al. [21]	1998	2003	Pointe-Noire	No limit
Nsimba et al. [22]	1999–2002	2004	Brazzaville, Pointe-Noire	0.5–<5 years
Nsimba et al. [23]	1999–2002	2005	Brazzaville, Pointe-Noire	0.5–<5 years
Mayengue et al. [24]	2003	2005	Brazzaville	≤5 years
Ndounga et al. [25]	2003–2004	2007	Brazzaville	<5 years
Ndounga et al. [13]	2003–2006	2008	Brazzaville	No limit
Van den Broek et al. [26]	2004	2006	Kindamba	0.5–<5 years
Mayengue et al. [27]	2005	2011	Brazzaville	No limit
Ndounga et al. [28]	2005	2013	Brazzaville	No limit
Pradine et al. [29]	2005–2006	2006	Pointe-Noire	1.4–17 years
Tsumori et al. [11]	2005–2006	2011	Brazzaville, Gamboma, Pointe-Noire	No limit
Oguike et al. [19]	2005–2006	2011	Brazzaville, Gamboma, Pointe-Noire	No limit
Moyen et al. [14]	2006	2010	Brazzaville	≤15 years
Ndounga et al. [30]	2006	2012	Brazzaville	0.5–10 years
Murai et al. [31]	2006	2015	Brazzaville, Gamboma, Pointe-Noire	No limit
Mita et al. [32]	2006	2016	Brazzaville, Gamboma, Pointe-Noire	No limit
Culleton et al. [12]	2007	2009	Pointe-Noire	No limit
Koekemoer et al. [33]	2009	2011	Boutoto	Not applicable
Koukouikila-Koussounda et al. [15]	2010	2012	Brazzaville	<10 years
Ibara-Okabande et al. [34]	2010–2011	2012	Brazzaville	<10 years
Ndounga et al. [35]	2010–2011	2015	Brazzaville	<10 years
Ossou-Nguiet et al. [18]	2011	2013	Brazzaville	0.4–14 years
Mbongo et al. [17]	2011–2013	2015	Brazzaville	15–39 years
Ntoumi et al. [9]	2011–2012	2013	Brazzaville, Pointe-Noire	≤15 years
Koukouikila-Koussounda et al. [36]	2012–2013	2015	Brazzaville	12–44 years
Ntoumi et al. [16]	2012–2013	2016	Brazzaville	12–44 years
Singana et al. [10]	2012–2013	2016	Owando	<12 years

**Table 2:** Main findings of studies that assessed the prevalence of malaria parasite infection

References	Period of sample collection	Year of publication	Asymptomatic infection		Infection in febrile patients		Severe malaria
			Microscopy (%)	PCR (%)	Microscopy (%)	PCR (%)	Microscopy (%)
Ndounga et al. [13]	2003–2006	2008	–	–	23.8^b,e^ versus 44.7^a,e^	–	–
Tsumori et al. [11]	2005–2006	2011	–	–	37^b,e^ versus 59^a,e^	42^b,e^ versus 75^a,e^	–
Moyen et al. [14]	2006	2010	–	–	–	–	14.7^b^
Koukouikila-Koussounda et al. [15]	2010	2012	8.6^a,c^	16^a,c^	–	–	–
Ossou-Nguiet et al. [18]	2011	2013	–	–		–	34.9^b^
Mbongo et al. [17]	2011–2013	2015	4.4^b,d^	–	–	–	–
Ntoumi et al. [9]	2011–2012	2013	–	–	12^b,c^ versus 17^b,d^	–	–
Ntoumi et al. [16]	2012–2013	2016	7^a,d^	19^a,d^	–	–	–

^a^Prevalence in sub-urban area

^b^Prevalence in urban areas

^c^Children

^d^Pregnant women

^e^No age limit

The study with the largest sample size was conducted from 2003 to 2006 in southern part of Brazzaville just before introduction of ACT in the country [13]. The authors found that in peri-urban area, of more than 1090 febrile patients examined, 44.7% had clinical malaria. Whereas in urban area, of 10,603 febrile patients examined, only 23.8% had clinical malaria. It was also observed that the pick of infections occurred between November and January and in March-April [13]. In the study conducted by Tsumori et al. [11] with samples collected in 2005–2006 from patients residing in urban and peri-urban areas of Brazzaville, Pointe Noire and Gamboma, plasmodial infection was screened by microscopy and by PCR. By microscopy, 37% of patients residing in urban areas were positive for *P. falciparum* compared to 59% of those residing in the peri-urban areas. With regard to PCR corrected results, the percentages rose to 42% *P. falciparum* positive for the urban residents and 75% positive for peri-urban residents, indicative a percentage of 11 and 20% of submicroscopic infections in these two populations, respectively. Another important study conducted before adoption of ACT was concomitantly done in different pediatric services of four hospitals in Brazzaville from January to August 2006 [14]. With the objective of assessing the real prevalence of severe malaria among young children in pediatric services, this study included more than 10,000 children. The overall severe malaria prevalence was 14.7%. Children of >5 years old were the most affected group as the rate reached 57.6%. The authors also determined 26.3% of death rate in this children population [14].

Following the replacement of chloroquine with ASAQ or AL in 2006 for the treatment of uncomplicated malaria, the first study that reported the prevalence of malaria parasite infection was conducted in 2010 in a peri-urban area of southern Brazzaville [15], an area which has been characterized as highly endemic with perennial malaria transmission [37]. In this study, from April to June 2010, 313 children below 10 years of age enrolled in a cohort for malaria surveillance were screened for *P. falciparum* asymptomatic carriage using microscopy and PCR as diagnostic techniques. The reported prevalence of infection was 8.6 and 16% by microscopy and PCR, respectively [15]. In a prospective and longitudinal study conducted from January 2011 to December 2013 at University Hospital Centre of Brazzaville, 13,883 pregnant women were screened by microscopy at delivery for malaria parasite carriage and newborns from positive mothers were further screened. A total of 610 mothers (4.4%) were found positive and 64% of newborns from these mothers also carried malaria parasites [17]. From October 2011 to February 2012, Ntoumi et al. carried out a malaria survey among under 15 years old children and pregnant women in different public health centers in Brazzaville and Pointe-Noire [9]. The main objective of the study was to document laboratory-confirmed cases of malaria using microscopy and/or rapid diagnostic tests (RDTs). *P. falciparum* was the only species detected and the prevalence of infections among more than 3000 children and 700 pregnant women ranged from 8 to 29, and 8 to 24%, respectively [9]. Another study by Ntoumi et al. using blood samples collected from March 2012 to December 2013 in an antenatal clinic located in peri-urban area of southern Brazzaville among pregnant women, reported a prevalence of asymptomatic *P. falciparum* infection at 7 and 19% when using microscopy and PCR, respectively [16]. A study by Ossou-Nguiet et al. conducted from July to December 2011 at the intensive care department of pediatric service of the University Hospital Center focused on determining the frequency and determinants of severe malaria among hospitalized children. A total of 1135 children were enrolled in this study and 34.9% (396) of them were diagnosed as severe malaria cases. Among them, 35.9% were further classified as coma cases, while 23.5, 20.8, 10.3, 7.4 and 3.6% were found to experience convulsions, coma and anaemia, prostration, convulsions and anaemia, and convulsions and respiratory distress, respectively [18].

**Fig. 3:**
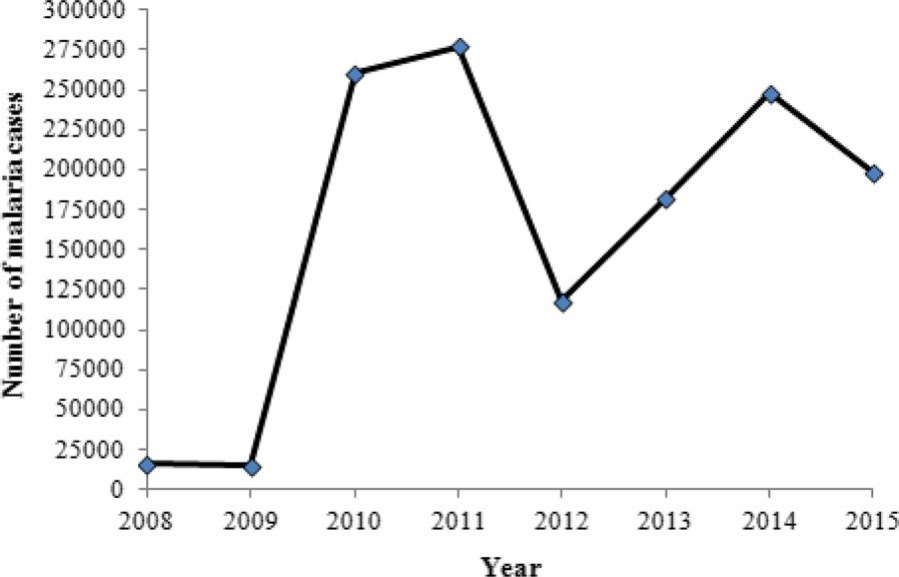
Number of malaria cases as reported by the National Malaria Control Program

Apart from the published articles, data available at the Ministry of Public Health and provided by the NMCP were also considered. In the national malaria report 2015 (released in 2016) [8] and the strategic plan for malaria control in the RoC from 2014 to 2018 (released in 2014) [5], the number of confirmed malaria cases recorded in public health centres countrywide from 2008 to 2015 were reported. Overall, malaria showed a fluctuation trend during the last 8 years (Fig. 3). The number of cases was 160,000 in 2008, 150,000 in 2009, 260,000 in 2010, 277,263 in 2011, 117,640 in 2012, 182,026 in 2013, 248,159 in 2014 and 198,047 in 2015 [5, 8]. As there is no such data for the years before ACT, it is difficult to give a comprehensive interpretation of this fluctuation.

### Malaria vectors

Malaria entomological studies are very limited in the RoC. From 1992 to June 2016, only one published investigation was found [33]. Conducted in 2009 in the village Boutoto located in the vicinities of Pointe-Noire, the study aimed to obtain the baseline vector information. *Anopheles gambiae sensu stricto* (s.s.) was the only mosquito species identified, and it had a high *P. falciparum* infection rate (9.6%). In this study, insecticide susceptibility was also assessed and the authors mentioned that multiple insecticide resistance was detected in this vector population with full susceptibility observed to only one insecticide class, the organophosphate [33].

In 2014, the NMCP documented entomological surveys conducted in 2013 by Antonio-Nkondjio and Bitsindu in the Bouenza department located at 300 km southern Brazzaville. *Anopheles gambiae s.l.* and *Anopheles funestus* were reported as the predominant vectors, while other species including *Anopheles coustani*, *Anopheles hancocki*, *Anopheles nili*, *Anopheles moucheti* and *Anopheles paludis* were found to be potential secondary vectors [5].

### Genetic diversity and multiplicity of *Plasmodium falciparum* in isolates from RoC

Number of information on the genetic diversity and the multiplicity of *P. falciparum* infections have been generated using samples from the RoC. A total of 8 published articles have been found, for which, different molecular markers have been used. The genes for the merozoite surface protein 1 and 2 have been used, *msp2* alone has been used in four investigations [9, 15, 16, 34], *msp1* and *msp2* in one study [27], and microsatellites in two studies with one utilizing *msp1* in addition to the microsatellite markers [11, 21].

In 2003, analysing 32 isolates of *P. falciparum* collected in 1998 at Pointe Noire, Durand et al. reported a high genetic diversity based on 28 microsatellites with a mean number of alleles per locus of 7.75 and the expected heterozygosity of 0.78 [21]. This trend was further confirmed by Tsoumori et al. who analysed microsatellite loci of *P. falciparum* isolates collected in urban and peri-urban areas in southern Brazzaville in 2005 [11]. In the study by Mayengue et al. [27], with 125 *P. falciparum* clinical isolates collected from children in south part of Brazzaville in 2005, the allelic specific *msp1* and *msp2* genotyping showed that malaria parasite population in Brazzaville is highly diverse. A total of 15 *msp1* and 20 *msp2* distinct alleles were identified. For *msp1*, K1 was found to be the predominant allelic type, whereas 3D7 family was the most prevalent for *msp2*. The overall mean MOI was 2.2 and 72% of the isolates carried more than one genotype [27]. In 2010, Four years after adoption of ACT in the country, Koukouikila-Koussounda et al. characterized the genetic polymorphism of *msp2* gene in *P. falciparum* isolates collected from Congolese children with asymptomatic infections [15]. Eighten different allele types were identified, suggestive of a high genetic diversity, in which, 8 belonged to the 3D7 family, which was found to be the predominant family, and 10 to FC27. However, the MOI and the rate of isolates with multiple genotypes were found to be low, 1.3 and 28%, respectively [15]. The cohort of children enrolled in this study was followed up for a year to monitor uncomplicated falciparum malaria episodes and isolates collected from some of the children who experienced uncomplicated malaria were later on analysed by Ibara-Okabando et al. [34] with regard to parasite diversity using *msp2* as molecular marker. A high genetic diversity of parasite population was observed with 21 different alleles detected (11 for 3D7 family and 10 for the FC27). Here, the MOI of 1.7 was determined and 54% the isolates harboured more than one *msp2* genotype [34].

Recent studies among children and pregnant women from Brazzaville and Pointe-Noire conducted in 2011–2012 by Ntoumi et al. [9] provided additional information on the genetic diversity and the MOI. The molecular characterization of the *msp2* gene revealed the presence of 11 (5 of 3D7 and 6 of FC27) and 22 alleles (13 of 3D7 and 9 of FC27) in *P. falciparum* isolates collected from 2011 to 2012 from children aged >15 years with uncomplicated malaria in Brazzaville and Pointe-Noire, respectively. The MOI was similar (about 1.7) in the two cities [9]. In another study, analysing isolates collected in 2012–2013 from pregnant women with asymptomatic *P. falciparum* infection using the same molecular marker, 11 alleles of the 3D7 family and 18 of the FC27 family were detected [16]. This is indicative of a higher genetic diversity in isolates from pregnant women that that of children. The rate of alleles belonging to the 3D7 and FC27 families was 62 and 38%, respectively. The authors also reported that 40.3% of the isolates harboured more than one *msp2* genotype and the overall MOI was 1.6 [16].

### Drug efficacy studies

In the period 1992 to 2005, before adoption of ACTs, six efficacy in vivo studies had been conducted, of which one was based on the 1973 WHO protocol for asymptomatic children to assess chloroquine efficacy [20], one on 14-day follow-up WHO protocol to assess chloroquine and sulfadoxine-pyrimethamine (SP) [22], and four on the current 28-day follow-up WHO protocol to evaluate therapeutic efficacy of chloroquine [24], SP [25], ASAQ, AL and AS + SP [26], AL and then ASAQ [28, 30]. The study based on the 1973 WHO protocol was conducted in 1993 in three southern regions of the country (Niari, Kouilou and Pool which included Brazzaville) and the authors reported that 7 days after the standard three-day treatment with chloroquine at 25 mg/kg, 20–60% of cases were still found to carry malaria parasites [20]. In the study using the 14-day follow-up WHO protocol and conducted in Brazzaville and Pointe-Noire from 1999 to 2002, the cure rate of chloroquine was 38.5% and that of SP was 95.8%. SP efficacy was further assessed in the second phase of this study and the cure rate of 100% was recorded [22]. However, studies carried out using the 28-day follow-up WHO protocol showed high level of treatment failure for chloroquine (95.7%) [24] and SP (31.2%) [25], while AL and ASAQ were found to be highly effective to treat uncomplicated malaria with the reported 28-day PCR-corrected cure rates of 96.9 [30] and 100% [26] for AL, and 94.4% [28] and 98.5% [26] for ASAQ. AS + SP, with the 28-day PCR-corrected cure rate of 90% was also effective but less than AL and ASAQ [26].

In 2006, the government of the RoC adopted a new treatment policy for uncomplicated malaria with ASAQ and AL as the first and second-line drugs, respectively [38]. However, in 2014, AL became the first- and ASAQ the second-line drug. Since 2006, two studies were conducted, one in Brazzaville and the other in Owando (located at 550 km to north of Brazzaville), to evaluate the efficacy of ASAQ and AL. The study in Brazzaville reported a PCR-corrected efficacy of 97% for ASAQ and 96.4% for AL [35]. In the study in Owando et al., the PCR-corrected efficacy was 100% for ASAQ and 98% for AL [10].

### *Plasmodium falciparum* in vitro resistance studies

Two studies have been conducted to assess the level of in vitro resistance of *P. falciparum* parasites to standard anti-malarial drugs. The isotopic test was used in the two studies. The first study was conducted in Brazzaville in 1993 and 34 *P. falciparum* isolates were tested with chloroquine, quinine and mefloquine. In addition, halofantrine was also tested on 35 *P. falciparum* isolates. The resistance rates were 61.8, 17.7, 3 and 0% for chloroquine, quinine, mefloquine and halofantrine, respectively [20]. The second investigation was done in Pointe-Noire where *P. falciparum* isolates were collected from March 2005 to January 2006 and their sensitivity assessed against 11 drugs: chloroquine, quinine, mefloquine, atovaquone, dihydroartemisinin, doxycycline, cycloguanil, lumefantrine, monodesethylamodiaquine, halofantrine and pyrimethamine [29]. The in vitro resistance rates were 75.5% for chloroquine, 68% for pyrimethamine, 36% for cycloguanil, 7% for mefloquine, 6% for quinine, 2% for monodesethylamodiaquine and 0% for the remaining drugs [29]. These studies contributed demonstrate evidence of high level of chloroquine resistance in the RoC. Since adoption of ACT in 2006, no in vitro study has been conducted on field isolates.

### *Plasmodium falciparum* drug resistance genes

A total of eight articles have been found reporting data on molecular markers of *P. falciparum* resistance to anti-malarials with samples collected from 1999 to 2015 [11, 15, 23–25, 31, 32, 36]. Overall, six molecular markers have been studied: the *P. falciparum* chloroquine resistance transporter (*pfcrt*) gene, associated with chloroquine resistance, the dihydrofolate reductase (*dhfr*) and the dihydropteroate synthase (*dhps*) genes, which are linked with pyrimethamine resistance and sulfadoxine resistance, respectively, the *P. falciparum* Klech-13 (K13) propeller gene, which has recently been linked with artemisinin resistance, and MAL10-688956 and MAL13-1718319 single nucleotide polymorphisms (SNPs) which have also recently been proposed as molecular markers of artemisinin resistance which is defined as a delayed clearance of *P. falciparum* parasites following ACT. The most analysed molecular markers are *pfcrt*, *dhfr* and *dhps* for which the main findings are summarized in Table 3.

**Table 3:** Main findings of studies that analysed *pfcrt, dhfr and dhps* genes

References	Period of sample collection	Year of publication	*pfcrt*	*dhfr*				*dhps*				*dhfr* and *dhps* (%)
			76T (%)	51I	59R	108 N	Triple	436A	437G	540E (%)	Triple (%)	
Nsimba et al. [23]	1999–2002	2005	97.1	82.4%	62.0%	86.6%	87.3%	17.5%	68.3%	0.0	–	73.0^a^/0.0^b^
Mayengue et al. [24]	2003	2005	98.0	–	–	–	–	–	–	–	–	–
Ndounga et al. [25]	2003–2004	2007	–	97.5	66.2	98.8	97.5	12.5	85.0	–	–	52.2^a^/6.2^b^
Tsumori et al. [11]	2005–2006	2011	88–97	–	–	–	50–68%	–	–	–	–	–
Koukouikila-Koussounda et al. [15]	2010	2012	92	–	–	–	–	–	–	–	–	–
Koukouikila-Koussounda et al. [36]	2012–2013	2015	–	88.0%	85.0%	79.1%	60.0%	67.1%	98.5%	55.2	>50	>50^b^/25.8^c^

^a^Quadruple mutants

^b^Quintuple mutants

^c^Sextuple mutants

The *pfcrt* gene has been studied in four articles and for all the K76T mutation was characterized. Nsimba et al. [23] with isolates collected in 1999 at Pointe-Noire and in 2001–2002 at Brazzaville reported a prevalence of 97.1% of *P. falciparum* isolates carrying the 76T mutation. This prevalence was similar to the rate of 98% reported by Mayengue et al. [24], 88–97% by Tsumori et al. [11], and 92% by Koukouikila-Koussounda et al. [15].

Molecular analysis of mutations associated with resistance to antifolates was performed by Nsimba et al. [23], Ndounga et al. [25], Koukouikila-Koussounda et al. [36], and Tsumori et al. [11] (who only analysed the *dhfr* point mutations). In all of these studies, *dhfr* point mutations at codons 51, 59 and 108 were found at high frequencies while the mutation at codon 164 was absent. For *dhps*, high prevalence of the mutation at codon 437 was observed in the three studies, while higher frequency of the mutation at codon 436 and the presence of mutation at codon 540 were only observed in the recent study, with samples collected from pregnant women in 2012–2013 at Brazzaville [36].

The K13 propeller gene was characterized by Mita et al. [32], and MAL10-688956 and MAL13-1718319 SNPs have been characterized by Murai et al. [31]. In both studies they used the same set of archived *P. falciparum* isolates collected in 2005–2006 in Brazzaville, Pointe-Noire and Gamboma, and none of the mutations associated with artemisinin resistance was found.

## Discussion

The purpose of this review is to provide an overview of published data and available information on malaria situation in RoC and to identify gaps in knowledge in order to contribute in research-based solutions adapted for the country. In the present review, as the first goal, we attempted to determine if there was any change in malaria epidemiology in RoC from 1992 to 2015, with regard to malaria parasites and vectors and in the presentation of the disease as well. The year 1992 was chosen because the first national policy for malaria control established by the NMCP was adopted that year. Besides, all the research institutions including those involved in malaria research were nationalized.

As a summary, during almost 24 years, a total of 28 studies published in peer reviewed journals were conducted the RoC in relation to different aspects of malaria infection. Concerning malaria burden, lower rates of malaria parasite infection in children and adults were observed in studies conducted from 2009 to 2015 compared to those conducted from 1992 to 2006 regardless of the diagnostic method (microscopy or PCR). This is suggestive of a decline malaria prevalence and incidence during the past 7 years and might be attributable to the scaling-up of malaria interventions in the country with the support of Global fund for HIV/AIDS, malaria and tuberculosis, including the use ACT which are freely provided to children of ≤15 years in public health facilities since 2008 associated with mass distribution of LLINs from 2008 to 2012. Further evidence on sustainability of the trend to the years later is important to ascertain such attributes.

With regard to *Plasmodium* species distribution, *P. falciparum* is by far the predominant malaria parasite occurring in the country accounting for almost 100% of malaria cases [11]. This observation on the extreme predominance of *P. falciparum* in the RoC is in accordance with findings from studies conducted in other Central African countries [39, 40].

The genetic diversity of *P. falciparum* was characterized trough different studies and the MOI was also determined. These two parameters are known to be good indicators of the level of premunition of populations living in endemic areas and correlate with the extent of the parasite population diversity as well as the transmission intensity [41, 42]. They are also important determinants of malaria control interventions. Overall, a significant diversity of *P. falciparum* population has been observed in all the studies and this is representative of areas with holo- or hyper-endemic malaria transmission. The High genetic diversity remains similar before and after introduction of ACT in 2006 and scaling up of control measures in the RoC. This implies that most of the parasite clones are still persisting and the interventions did not have impact on specific *P. falciparum* clones. Lower values of the MOI (1.3–1.7) have been obtained in studies conducted after these interventions than those conducted before (2.2–2.3). This trend, in addition to low rates of parasite carriage observed in studies conducted after 2006 may suggest that the implemented control measures have resulted in a substantial decrease of transmission. However, this needs to be assessed by conducting entomological studies that allow determination of the EIR in each area, which correlate well with the level of malaria transmission.

Vector control is one of the main interventions for an effective malaria control programme. To be successful, this intervention needs to rely on availability of data on vector population, level of transmission and insecticide resistance. Unfortunately, only one publication was found [33] and this reflects a major gap in knowledge about the malaria situation in RoC. In that study, 523 *An. gambiae* complex specimens were collected and the specimen identification revealed that all were *An. gambiae s.s.*, of which, 95.4% were further molecularly characterized as the S-form. Importantly, these vectors were found to be highly resistant to multiple insecticide classes. This review would classify entomological research as a key priority in the country. Moreove, as *An. funestus*, *An. coustani* and *An.hancocki* were found to be potential minor malaria vectors [5], their implication in malaria transmission and the level of resistance to insecticides have yet to be further elucidated.

Since the utilization of ACT for the treatment of uncomplicated malaria, monitoring studies are required to detect any emergence of artemisinin resistant strains as it has been already reported from South East Asia [43, 44]. Overall in vivo efficacy studies conducted so far provided evidence of good efficacy of currently recommended ACT in RoC. It is observed that only one study analysed mutations on *P. falciparum* K13 propeller gene in isolates from RoC [32], and it would be important that local scientists screen regularly isolates from different parts of the country. Chloroquine and SP were banned for the treatment of uncomplicated malaria in the RoC since 2006 due to high level of parasite resistance. Studies conducted five to seven years after their withdrawal still show high rates of parasites carrying mutations associated with resistance to these two molecules. This would like to suggest that these molecules remain inactive, despite the fact that SP is still used as intermittent preventive treatment in pregnant women. Moreover, the presence of the 540 *dhps* mutation (in *P. falciparum* isolates collected in 2011–2012) [36], which was absent before SP withdrawal [23], and higher level of quintuple *dhfr*/*dhps* mutations might suggest that SP resistance become more pronounced.

As a second key priority highlighted by this review is the limited number of publications on malaria in pregnancy in RoC. For instance, there is no data on malaria parasites collected from pregnant women before the introduction of ACT and the first data were published in 2013 [9]. The researchers from Congo working on malaria are also involved in the Central Africa Network on Tuberculosis, HIV/AIDS and Malaria (CANTAM) which aims at building capacities for the conduct of clinical trials. Therefore, it would be of interest to test alternative drugs for preventing malaria during pregnancy in Congolese women.

## Conclusion

A total of 28 peer reviewed articles and two official documents from the NMCP were included in this review and have shown that malaria is still endemic in the country. Unfortunately, the majority of studies were conducted in Brazzaville followed by Pointe-Noire (the two main cities). Therefore, results cannot formally be generalized. We note two major positive points: ASAQ and AL are highly efficacious in treatment of uncomplicated malaria and there is substantial reduction of malaria transmission since introduction of ACTs in the country. However, a strong resistance to SP is observed in parasites collected from Congolese pregnant women.

## Abbreviations

WHO: World Health Organization
RoC: Republic of Congo
EIR: entomological inoculation rate
ACT: artemisinin-based combination therapy
AL: artemether–lumefantrine
ASAQ: artesunate–amodiaquine
IPTp-SP: intermittent preventive treatment with sulfadoxine–pyrimethamine for pregnant women
LLIN: long-lasting insecticide-treated net
NMCP: National Malaria Control Programme
MHP: Ministry of Health and Population
MeSH: medical subject heading
PCR: polymerase chain reaction
RDT: rapid diagnostic test
msp2: merozoite surface protein 2 gene
msp1: merozoite surface protein 1 GENE
MOI: multiplicity of infection
pfcrt: *Plasmodium falciparum* chloroquine resistance transporter
dhfr: dihydrofolate reductase
dhps: dihydropteroate synthase
K13: Klech-13
SNP: single nucleotide polymorphism
HIV/AIDS: human immunodeficiency virus/acquired immune deficiency syndrome
CANTAM: Central Africa Network on Tuberculosis, HIV/AIDS and Malaria
